# Hyperexpression of α-hemolysin explains enhanced virulence of sequence type 93 community-associated methicillin-resistant *Staphylococcus aureus*

**DOI:** 10.1186/1471-2180-14-31

**Published:** 2014-02-10

**Authors:** Kyra YL Chua, Ian R Monk, Ya-Hsun Lin, Torsten Seemann, Kellie L Tuck, Jessica L Porter, Justin Stepnell, Geoffrey W Coombs, John K Davies, Timothy P Stinear, Benjamin P Howden

**Affiliations:** 1 Department of Microbiology and Immunology, University of Melbourne, Melbourne, Victoria 3052, Australia; 2 Department of Microbiology, Monash University, Clayton, Victoria 3800, Australia; 3 Austin Centre for Infection Research (ACIR), Infectious Diseases Department, Austin Health, PO Box 5555, Heidelberg, Victoria 3084, Australia; 4 Microbiology Department, Austin Health, Heidelberg, Victoria 3084, Australia; 5 Victorian Bioinformatics Consortium, Monash University, Clayton, Victoria 3800, Australia; 6 School of Chemistry, Monash University, Clayton, Victoria 3800, Australia; 7 Australian Collaborating Centre for Enterococcus and Staphylococcus Species (ACCESS) Typing and Research, PathWest Laboratory Medicine-WA, Royal Perth Hospital, Perth, Western Australia 6000, Australia; 8 School of Biomedical Sciences, Curtin University, Bentley, Western Australia 6102, Australia

**Keywords:** *Staphylococcus aureus*, CA-MRSA, Pathogenesis, Alpha-hemolysin

## Abstract

**Background:**

The community-associated methicillin-resistant *S. aureus* (CA-MRSA) ST93 clone is becoming dominant in Australia and is clinically highly virulent. In addition, sepsis and skin infection models demonstrate that ST93 CA-MRSA is the most virulent global clone of *S. aureus* tested to date. While the determinants of virulence have been studied in other clones of CA-MRSA, the basis for hypervirulence in ST93 CA-MRSA has not been defined.

**Results:**

Here, using a geographically and temporally dispersed collection of ST93 isolates we demonstrate that the ST93 population hyperexpresses key CA-MRSA exotoxins, in particular α-hemolysin, in comparison to other global clones. Gene deletion and complementation studies, and virulence comparisons in a murine skin infection model, showed unequivocally that increased expression of α-hemolysin is the key staphylococcal virulence determinant for this clone. Genome sequencing and comparative genomics of strains with divergent exotoxin profiles demonstrated that, like other *S. aureus* clones, the quorum sensing *agr* system is the master regulator of toxin expression and virulence in ST93 CA-MRSA. However, we also identified a previously uncharacterized AraC/XylS family regulator (AryK) that potentiates toxin expression and virulence in *S. aureus*.

**Conclusions:**

These data demonstrate that hyperexpression of α-hemolysin mediates enhanced virulence in ST93 CA-MRSA, and additional control of exotoxin production, in particular α-hemolysin, mediated by regulatory systems other than *agr* have the potential to fine-tune virulence in CA-MRSA.

## Background

Community-associated methicillin-resistant *Staphylococcus aureus* (CA-MRSA) is an emerging global problem with very similar clinical presentations across different clones*,* despite significant genetic diversity [1]. Many CA-MRSA strains carry *lukSF-PV* in the accessory genome, which encodes the Panton-Valentine leukocidin (PVL), an exotoxin that causes neutrophil lysis [1]. Although there has been considerable controversy as to the role of this toxin in CA-MRSA pathogenesis, some of this may be explained by a variable, species dependent susceptibility to PVL – human and rabbit neutrophils are lysed by PVL at very low concentrations whilst mouse and monkey neutrophils are less susceptible, making the interpretation of animal model data difficult in some cases [2]. Additionally, the importance of PVL is also likely to be dependent on the site of infection. In the rabbit pneumonia model, PVL has been demonstrated to have a clear role in mediating severe lung necrosis and inflammation [3]. In contrast, in skin infection, even in the rabbit model, its role remains less clear [4,5].

Notwithstanding PVL, the increased expression of other core genome virulence determinants also contributes significantly to the increased virulence of CA-MRSA strains [6,7]. These include α-hemolysin (Hla) and α-type phenol soluble modulins (PSMs). Hla is a pore-forming exotoxin that lyses many cells including red cells, platelets, monocytes and endothelial cells [8]. Hla has been demonstrated to be an important mediator of virulence in skin infection and pneumonia [9,10]. The α-type PSMs have been recently characterized and they lyse neutrophils and red cells [11,12]. The α-type PSMs also mediate virulence in skin infection and septicemia and of these, PSMα3 is the most potent [11].

The study of unique, distantly related CA-MRSA clones that also demonstrate enhanced virulence, may provide insights into the emergence of the global CA-MRSA phenomenon, and also help define the genomic determinants of enhanced virulence. In Australia, the singleton ST93 CA-MRSA clone ST93 ("Queensland clone") has become dominant in the community [13], and we and others have demonstrated that our reference ST93 strain JKD6159 was highly virulent and caused severe skin infection in a mouse model compared to other CA-MRSA strains including USA300 [14,15].

In this study we used exotoxin analysis, functional genomics and a murine infection model to investigate the relative contribution of α-hemolysin, α-type phenol soluble modulins and Panton-Valentine leukocidin to the enhanced virulence of ST93 CA-MRSA. We show that increased virulence in the BALB/c mouse skin infection model is less dependent on α-type phenol soluble modulin or Panton-Valentine leukocidin production but is instead due to high-level expression of α-hemolysin in this clone, controlled predominantly by the *agr* system.

## Results and discussion

The emergence of CA-MRSA is a major public health issue, and there is a clear need to understand the basis for both virulence and transmission of global clones of CA-MRSA. The genetically distinct CA-MRSA clone ST93-IV [2B] has rapidly become the dominant clone in Australia and its rise accounts for the increase in incidence of CA-MRSA as a whole in this country [13]. We, and others have previously shown that ST93 strain JKD6159 is the most virulent global clone of *S. aureus* in murine models [14,15]. To determine the mediators of virulence in this clone we initially studied exotoxin expression in a large collection of ST93 *S. aureus* from around Australia, and compared representative high and low expressing strains to an international selection of clones.

### Exotoxin expression in ST93 CA-MRSA strains

**Figure 1:**
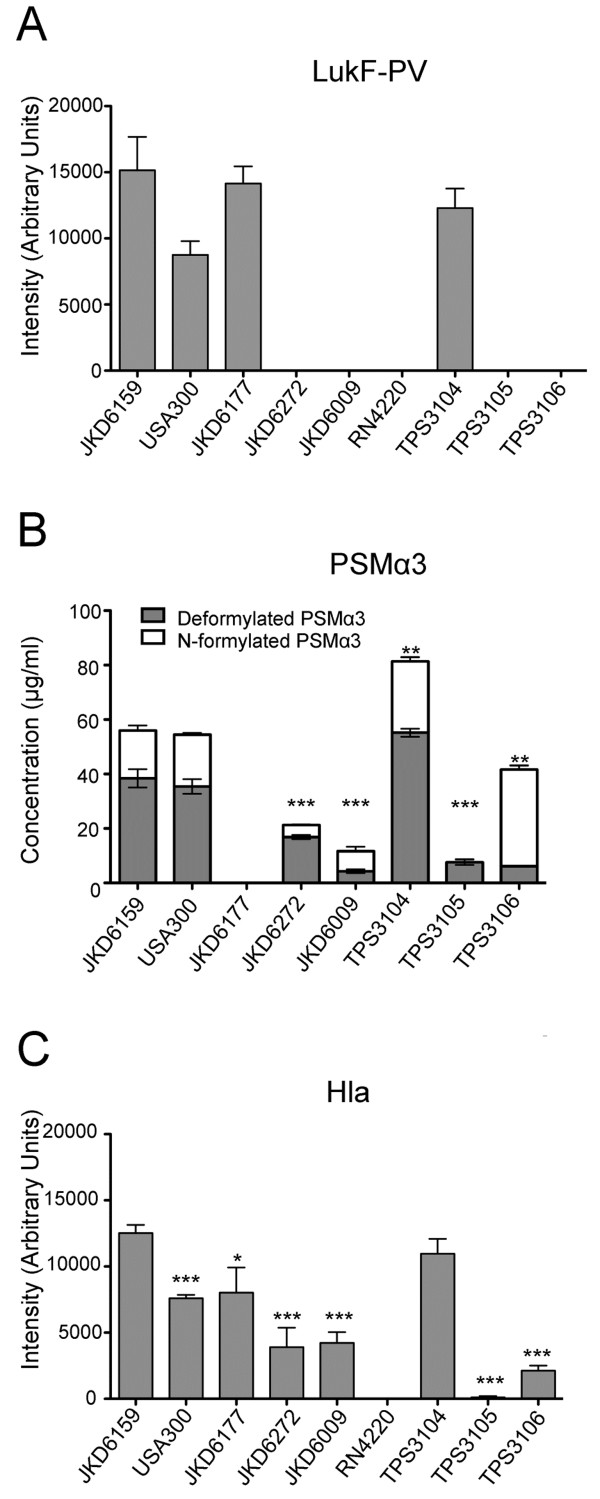
***In vitro *exotoxin expression of wildtype *S. aureus *isolates.** JKD6159 (ST93-IV [2B]) compared with non-ST93 CA-MRSA strains FPR 3757 USA300 (ST8-IV [2B]), JKD6177 (ST30-IV [2B]), and JKD6272 (ST1-IV [2B]); Hospital-associated MRSA strain JKD6009 (ST239-III [3A]), wildtype ST93 strains TPS3104 (ST93-IV [2B]), TPS3105 (ST93-IV [2B]), and TPS3106 (ST93-V [5C2&5]). **(A)** LukF-PV expression measured by quantitative Western blot. RN4220 was included as a negative control because it does not contain *lukF-PV*. All PVL negative strains did not express LukF-PV*.* There was no significant difference in the amount of LukF-PV expressed by the *S. aureus* strains containing *lukSF-PV*. Data shown are mean intensity of bands in arbitrary units and SEM. **(B)** PSMα3 expression measured by HPLC. JKD6177 did not produce PSMα3. JKD6272 (p = 0.0003), JKD6009 (p = 0.0003), TPS3105 (p < 0.0001) and TPS3106 (p = 0.0100) produced less deformylated and N-formylated PSMα3 compared to JKD6159. There was no difference between PSMα3 production by JKD6159 and USA300. TPS3104 expressed more PSMα3 than JKD6159 (p = 0.0029). Data shown are mean concentration (μg/ml), presented as vertical stacked bars and SEM. Deformylated PSMα3 is shown in grey bars. N-formylated PSMα3 is shown in white bars. **(C)** Hla expression measured by quantitative Western blot. RN4220 was included as a negative control because it does not express Hla. JKD6159 expressed more Hla compared to all non-ST93 wildtype strains (p < 0.0001 for all strains except JKD6177 p = 0.0107). TPS3105 and TPS3106 produced significantly less Hla (p < 0.0001). There was no difference in Hla production between JKD6159 and TPS3104. Data shown are mean intensity of bands in arbitrary units and SEM. Note, ***p < 0.001, **p < 0.01, *p < 0.05.

*Staphylococcus aureus* expresses a wide range of exotoxins that may contribute to virulence. Because Hla, PVL and α-type PSMs have been found by others to be important virulence factors in CA-MRSA strains [9,11,16], we measured *in vitro* expression of these exotoxins by the wildtype ST93 strains and non-ST93 comparator strains. The main isolates used in this study are described in Table 1, while the collection of ST93 isolates from around Australia used for comparative exotoxin expression is from a study by Coombs *et al.*[17] and summarized in Additional file 1. The comparison of expression of international clones to the ST93 reference strain JKD6159 and three additional ST93 strains selected for genome sequencing (see below) are shown in Figure 1, while the results for all 59 ST93 isolates compared to USA300 are shown in Additional file 2 (α-type PSMs) and Additional file 3 (Hla). The results of PVL analysis for the ST93 collection has been previously reported [17]. Because PVL is a 2-component exotoxin and both LukS-PV and LukF-PV are required for activity, we chose to measure LukF-PV expression by quantitative Western blot. LukF-PV was chosen over LukS-PV to obtain anti-LukF-PV antibody with increased specificity of binding as there was more sequence divergence between *lukF-PV* and the orthologous 2-component *S. aureus* exotoxins compared to *lukS-PV*. Although there are four α-type PSMs, PSMα3 causes the most significant neutrophil lysis [11] and we measured deformylated and N-formylated PSMα3 expression by high performance liquid chromatography (HPLC). The proportion of deformylated and formylated forms of PSMs depends on the growth conditions, the activity of staphylococcal peptide deformylase and strain background [7,18].

**Table 1:** Bacterial strains used in this study

***S. aureus *** **strain**	**Molecular type**	**Date of isolation**	**Place of isolation**	**Site of isolation**	**Relevant characteristics**	***lukSF-PV***	**reference**
***Clinical isolates***							
JKD6159	ST93-IV [2B]	2004	Victoria, Australia	Blood	Dominant Australian CA-MRSA clone	+	[14]
TPS3104	ST93-IV [2B]	2009	Western Australia, Australia	Nasal cavity	Dominant Australian CA-MRSA clone	+	This study
TPS3105	ST93-IV [2B]	2005	New South Wales, Australia	Blood	Australian CA-MRSA clone	-	This study
TPS3106	ST93-V [5C2&5]	2008	Western Australia, Australia	Nasal cavity	Australian CA-MRSA clone	-	This study
JKD6272	ST1-IV [2B]	2002	Victoria, Australia	Blood	Australian CA-MRSA clone	-	[14]
JKD6260	ST1-IV [2B]	2008	Western Australia, Australia	Skin	Australian CA-MRSA clone	+	[14]
JKD6177	ST30-IV [2B]	2003	Melbourne, Australia	Blood	Australian CA-MRSA clone	+	[14]
FPR3757 USA300	ST8-IV [2B]	NA	San Francisco, USA	Wrist abscess	Dominant North American CA-MRSA clone	+	[19]
JKD6009	ST239-III [3A]	2002	New Zealand	Wound	Dominant Australian hospital-associated MRSA clone, AUS2/3	-	[20]
***Mutant strains***							
JKD6159∆*lukSF-PV*	ST93-IV [2B]				Isogenic unmarked *lukSF-PV* KO of JKD6159	-	This study
JKD6159∆*hla*	ST93-IV [2B]				Isogenic unmarked *hla* KO of JKD6159. Deletion encompassed genome coordinates 1121291–1120441.	+	This study
JKD6159∆*hla r*	ST93-IV [2B]				Isogenic unmarked *hla* KO repaired in JKD6159∆*hla*. Introduction of a novel PstI site within *hla*.	+	This study
JKD6159∆*psmα*	ST93-IV [2B]				Isogenic unmarked *psm-α* KO in JKD6159. Deletion encompassed genome coordinates 453364–45378.	+	This study
JKD6159∆*psmα r*	ST93-IV [2B]				Isogenic unmarked *psm-α* KO repaired in JKD6159∆*psm-α*. Introduction of a novel SalI site within *psm-α*	+	This study
JKD6159∆00043	ST93-IV [2B]				Isogenic unmarked SAA6159_00043 KO of JKD6159. Deletion encompassed genome coordinates 53156 - 54561	+	This study
JKD6159_AraC*r*	ST93-IV [2B]				Isogenic AraC/XylS regulator repaired in JKD6159	+	This study
TPS3105*r*	ST93-IV [2B]				Isogenic *agrA* repair of TPS3105	-	This study

KO: knockout, NA: not available.

### PVL

As previously reported [17], PVL expression was consistent across most ST93 strains. We found that there was no significant difference in the LukF-PV expression in the PVL positive strains JKD6159, TPS3104, USA300 and JKD6177. Although USA300 appeared to produce less LukF-PV than JKD6159, the difference was not statistically significant (p = 0.0943, Figure 1A).

### PSMα3

We found that the deformylated form of PSMα3 was almost always more abundant than the N-formylated form (Figure 1B and Additional file 2). The ST30 CA-MRSA strain JKD6177 did not produce any PSMα3. There was no significant difference in PSMα3 expression between JKD6159 compared to USA300, however JKD6159 produced more PSMα3 compared to JKD6272 (p = 0.0003) and JKD6009 (p = 0.0003). Compared to the other ST93 MRSA strains, JKD6159 produced more PSMα3 compared to TPS3105 (p < 0.0001), and TPS3106 (p = 0.01) but less than TPS3104 (p = 0.0029) (Figure 1B). Expression levels across the whole ST93 collection were variable, although many isolates produced levels at least equivalent to USA300 (Additional file 2).

### Hla

Hla expression appeared high for the majority of ST93 isolates, with the exception of four strains where expression was low (Additional file 3). JKD6159 produced greater levels of Hla than all the wildtype strains, including USA300 (p < 0.0001 for all strains except JKD6177, p = 0.0107, Figure 1C). There was no difference in Hla expression between JKD6159 and TPS3104.

Here we have demonstrated that the majority of ST93 strains consistently produce higher levels of Hla compared to other clones, including USA300, while production of PVL and α-type PSM is similar, suggesting that enhanced expression of Hla may be responsible for increased virulence of ST93 CA-MRSA.

### Comparative virulence of ST93 isolates with differential exotoxin expression

**Figure 2:**
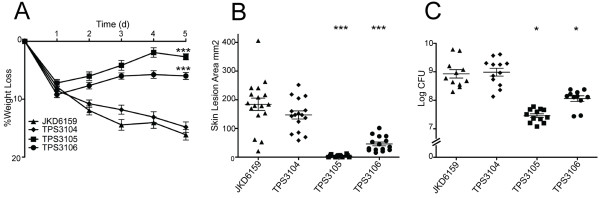
**Virulence characteristics of wildtype ST93 CA-MRSA isolates.** *S. aureus* JKD6159 compared with three other wildtype ST93 CA-MRSA isolates, TPS3104, TPS3105 and TPS3106 in a BALB/c mouse skin infection assay. At least 10 mice were used for each bacterial strain. **(A)** Weight loss induced by intradermal infection with *S. aureus* strains is demonstrated as percentage loss of weight over 5 days. The difference in percentage weight loss between JKD6159 and TPS3105 and TPS3106 was significant (p < 0.0001). There was no difference in weight loss between JKD6159 and TPS3104. Data shown are mean weight loss and SEM. **(B)** Skin lesion area (mm^2^) at 5 days after infection was significantly greater with JKD6159 infected mice compared to TPS3105 and TPS3106 (p < 0.0001). There was no difference in lesion area between JKD6159 and TPS3104. Data shown are mean area and SEM. **(C)** Recovery of *S. aureus* (log CFU) from infected tissues at 5 days after infection from JKD6159 infected mice was greater than with TPS3105 (p = 0.0177) and TPS3106 infected mice (p = 0.0328). There was no difference between JD6159 and TPS3104 infected mice. Data shown are mean CFU and SEM. Note, ***p < 0.001, *p < 0.05.

To further examine the role of these selected exotoxins in our mouse skin infection assay, we compared the virulence of four ST93 isolates selected based on their exotoxin expression profiles (high exotoxin expression, JKD6159 and TPS3104; low exotoxin expression, TPS3105 and TPS3106). TPS3104 was as virulent as JKD6159 in the mouse model in all outcome measures (Figure 2). In contrast, the strains with reduced exotoxin expression TPS3105 and TPS3106 were significantly less virulent compared to JKD6159, with less weight loss at day 5 of infection (p < 0.0001), smaller lesion size (p < 0.0001) and less CFU recovery from lesions (TPS3105, p = 0.0177; TPS3106, p = 0.0328) in the model (Figure 2).

### Impact of exotoxin expression on virulence of ST93 CA-MRSA in the murine skin infection model

To further characterize the contribution of each of the exotoxins to disease in the murine model, genetic deletion and complementation experiments were performed for each of the selected toxins.

### Hla

**Figure 3:**
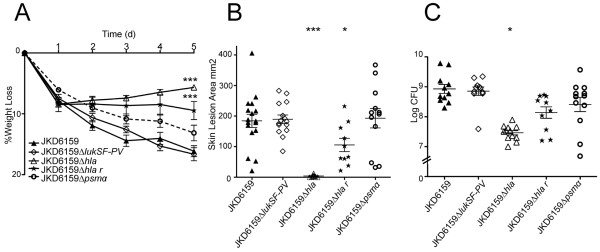
**Virulence characteristics of *S. aureus *JKD6159 and isogenic exotoxin mutants derived from JKD6159.** JKD6159 compared to isogenic PVL knockout (JKD6159∆*lukSF-PV*), isogenic Hla knockout (JKD6159∆*hla*), isogenic Hla complemented strain (JKD6159∆*hla r*) and isogenic PSM-α knockout (JKD6159∆*psmα*) in a BALB/c mouse skin infection assay. **(A)** Weight loss induced by intradermal infection with *S. aureus* strains is demonstrated as percentage loss of weight over 5 days. There was no significant difference between JKD6159, JKD6159∆*lukSF-PV* and JKD6159∆*psmα* infected mice. There was significantly less weight loss in mice infected with JKD6159∆*hla* compared to JKD6159 (p < 0.0001). There was also less weight loss in mice infected with JKD6159∆*hla* compared to JKD6159∆*hla r* (p = 0.0063). Mice infected with JKD6159∆*hla r* had less weight loss compared to JKD6159 (p = 0.0004). Data shown are mean weight loss and SEM. **(B)** There was no difference in skin lesion area (mm^2^) at 5 days after infection in mice infected with JKD6159 and JKD6159∆*lukSF-PV* and JKD6159∆*psmα.* Mice infected with JKD6159∆*hla* had significantly smaller lesions (p < 0.0001). In some mice, there was no cutaneous lesion seen. There were significantly smaller lesions in mice infected with JKD6159∆*hla* compared to JKD6159∆*hla r* (p < 0.0001). Mice infected with JKD6159∆*hla r* had smaller lesions compared to JKD6159 (p = 0.024). Data shown are mean area and SEM. **(C)** Recovery of *S. aureus* (log CFU) from infected tissues at 5 days after infection from JKD6159 infected mice was no different to that from JKD6159∆*lukSF-PV*, JKD6159∆*psmα* and JKD6159∆*hla r.* There was significantly less *S. aureus* recovered from JKD6159∆*hla* infected mice (p = 0.0177). There was also significantly less *S. aureus* recovered from JKD6159∆*hla* infected mice compared to JKD6159∆*hla r* (p = 0.0018). Data shown are mean CFU and SEM. Note, ***p < 0.001, *p < 0.05, compared to JKD6159.

Given the increased *in vitro* expression of Hla by JKD6159 and TPS3104 and the apparent correlation of this increased expression with increased virulence in the mouse skin infection model, we generated JKD6159∆*hla* and assessed this mutant in the mouse skin infection assay (Figure 3). There was a marked attenuation in virulence in all outcome measures with significantly decreased weight loss (p < 0.0001), lesion size (p < 0.0001) and CFU recovery (p = 0.0177). To confirm that an unintentional mutation introduced during the procedure to knock-out *hla* was not responsible for the reduced virulence in this strain, complete genome sequencing of the strain using Ion Torrent sequencing was performed. Mapping of sequence reads from JKD6159∆*hla* against JKD6159 (40× genome coverage) demonstrated no additional differences between JKD6159 and JKD6159∆*hla.* We also restored Hla function in this strain by using allelic replacement to repair the deletion in *hla* and create strain JKD6159∆*hla r*. As expected, Hla expression was absent in JKD6159∆*hla* and expression was restored in JKD6159∆*hla r* when tested by Western Blot (Additional file 4A)*.* JKD6159∆*hla r* also reverted to high virulence in the mouse skin infection assay (Figure 3). The apparent slight reduction in virulence of this *hla* repaired strain compared to wild type JKD6159 is explained by incomplete penetration of the restored *hla* allele in JKD6159∆*hla r*, resulting in mixed bacterial populations and reversion to JKD6159∆*hla* for some of the mice (Additional file 4B and C).

### α-type PSMs

In order to determine the contribution of α-type PSMs to virulence of JKD6159, we generated JKD6159∆*psmα* (deletion of the whole α-type PSM locus) and assessed this mutant in the mouse skin infection assay (Figure 3). There was no significant difference in virulence in all outcome measures; weight loss (p = 0.06), lesion size (p = 0.8174) and CFU recovery (p = 0.1925). The absence of PSMα3 production was verified by HPLC and the integrity of the JKD6159∆*psmα* was confirmed by complementation (Additional file 5). It is noteworthy that the level of expression of PSMα3 by JKD6159 was similar to USA300 (Figure 1), a strain that produces high levels of PSMs and where a contribution to virulence has been demonstrated [7,11]. Despite this, the deletion mutant (JKD6159∆*psmα*) demonstrated no attenuation of virulence compared to JKD6159 (Figure 3). The significantly divergent genetic background of ST93 compared with USA300 may account for this difference in the importance of α-type PSMs to the virulence of JKD6159 [6].

### PVL

We constructed an isogenic PVL negative mutant in JKD6159 by deleting *lukSF-PV*. Western Blot analysis confirmed the absence of LukF-PV in the mutant (Additional file 6). Assessment of the JKD6159Δ*lukSF-PV* mutant in the mouse skin infection model showed no decrease in virulence (Figure 3). Therefore PVL was not contributing to the increased virulence in JKD6159 in this murine model. Murine neutrophils, unlike rabbit and human neutrophils are relatively resistant to the effects of PVL so it is difficult to draw firm conclusions as to the human importance of this result [2]. However, the aim of this study was to uncover the mechanisms for the observed increased virulence of ST93 previously demonstrated using this mouse model [14]. Our results reinforce the results of others who have examined different *S. aureus* clones which indicate that Hla, rather than PVL is the main mediator of virulence in CA-MRSA in a mouse skin infection model [9,10,21,22]. It should be noted that other authors have concluded that the rabbit skin infection model gave very similar results to the mouse model for infection at the same site [4]. Nonetheless, testing of our PVL deletion mutant in a rabbit model may be warranted in future.

### Genome sequencing of three additional ST93 isolates

**Figure 4:**
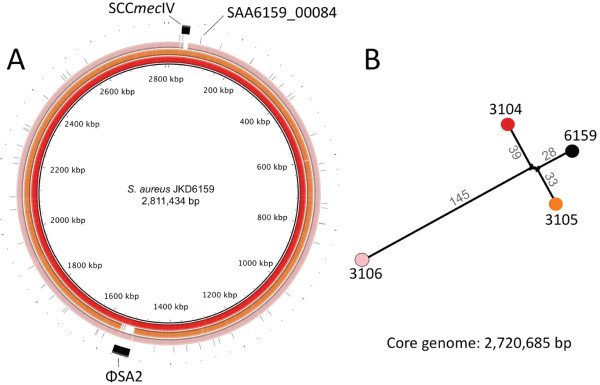
**Whole genome sequence analysis and comparison of JKD6159 with other ST93 CA-MRSA isolates. (A)** Circular diagram of the JKD6159, TPS3104, TPS3105 and TPS3106 chromosomes (from inner to outer circles). TPS3104, TPS3105 and TPS3106 contigs were mapped by BLASTN to JKD6159. TPS3104 contained SCC*mec*IV and ϕSA2 with *lukSF-PV*; TPS3105 contained SCC*mec*IV but lacked ϕSA2 and *lukSF-PV*; TPS3106 contained SCC*mec*V, and ϕSA2 without *lukSF-PV*. **(B)** ST93 *S. aureus* phylogeny inferred by split decomposition analysis from pairwise comparisons of the 253 variable nucleotide positions identified from the ST93 core chromosome of 2,720,685 bp. Figures indicate the number of nucleotide substitutions per branch. All nodes have 100% bootstrap support.

We have previously fully sequenced and annotated the genome of ST93 strain JKD6159 [14,23]. The differential virulence and exotoxin expression of some ST93 isolates compared to JKD6159 was then exploited by using whole genome sequencing and comparative genomics to determine the genetic basis for exotoxin expression in this clone. We selected the high expression strain TPS3104 and the low virulence and expression strains TPS3105 and TPS3106 to compare to JKD6159. *De novo* assembly of each of these strains resulted in ~700 contigs per isolate, with a genome length of 2.8 Mbp. The *de novo* assembly metrics are summarized in Additional file 7. The contigs were aligned to JKD6159 using BLASTN, with some important differences demonstrated between the strains (Figure 4A). TPS3104 contained SCC*mec*IV and ϕSA2 with *lukSF-PV*; TPS3105 contained SCC*mec*IV but lacked ϕSA2 and *lukSF-PV*; TPS3106 contained SCC*mec*V, and ϕSA2 without *lukSF-PV*. In addition, read-mapping against the complete JKD6159 genome (chromosome and plasmid: 2,832,164 bp) was then employed to define a 2,720,685 bp core genome among these four isolates (96% of the JKD6159 genome), revealing 253 polymorphic nucleotide positions, some of which were common to two strains. A phylogeny was inferred that confirmed the close relationship among all isolates, with TPS3106 more distantly related to the others (Figure 4B). The unmapped reads from each isolate were also subjected to *de novo* assembly to identify DNA not present in JKD6159. TPS3104 and TPS3105 contained no new sequences, while TPS3106 contained 34 kb of additional DNA, predominantly spanning the SCC*mec*V region.

### Comparative genomics of ST93 and the importance of *agr* in the virulence of ST93 CA-MRSA

We next explored the contribution of specific mutations to the differential virulence of the ST93 strains. Using our read mapping approach described above, we compared the genome sequences of TPS3104, TPS3105 and TPS3106 with each other and with JKD6159. There were a number of single nucleotide polymorphisms (SNPs) and insertions and deletions (indels) differentiating the strains from JKD6159 (Additional files 8, 9, 10). We searched for mutations in regulatory genes that could potentially explain the different virulence phenotypes of the strains. Notably, both avirulent ST93 strains, TPS3105 and TPS3106 contained mutations within the *agr* locus. We have since completed whole genome sequencing of TPS3151 and TPS3161 and found they contain predicted amino acid substitutions in AgrC that might disrupt *agr* function (Stinear *et al*., submitted). These isolates demonstrated low expression of Hla (Additional file 3).

Additionally, TPS3106 also contained a mutation in a gene encoding a previously uncharacterized AraC/XylS family regulatory protein. This was also of particular interest as members of this class have been shown to contribute to the regulation of exotoxin expression [24,25].

**Figure 5:**
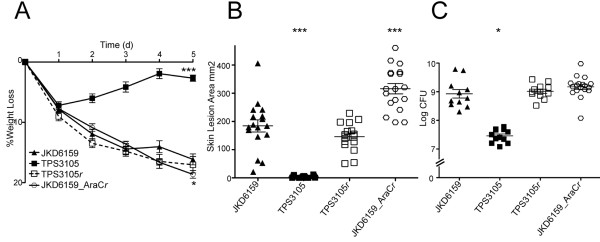
**The importance of *agr *and *aryK *in the virulence of ST93 CA-MRSA.** Isogenic repaired *agr* mutant TPS3105*r* compared to TPS3105 and JKD6159, and JKD6159 compared with isogenic repaired AraC/XylS family regulator mutant (JKD6159_AraC*r*) in a BALB/c mouse skin infection assay. At least 10 mice were used for each bacterial strain. **(A)** Weight loss induced by intradermal infection with *S. aureus* strains is demonstrated as percentage loss of weight over 5 days. There was no difference between JKD6159 and TPS3105*r* in all outcome measures. TPS3105*r* infected mice had significantly increased weight loss compared to TPS3105 (p < 0.0001). There was a small increase in weight loss in mice infected with JKD6159_AraC*r* compared to JKD6159 (p = 0.0311). Data shown are mean weight loss and SEM. **(B)** Skin lesion area (mm^2^) at 5 days after infection in TPS3105*r* infected mice was significantly increased compared to TPS3105 (p < 0.0001). Mice infected with JKD6159_AraC*r* had increased lesion area compared with JKD6159 (p < 0.0001). Data shown are mean area and SEM. **(C)** Recovery of *S. aureus* (log CFU) from infected tissues at 5 days after infection from TPS3105r was significantly greater than from TPS 3105 infected mice (p < 0.0001). There was no difference in *S. aureus* recovered from mice infected with JKD6159 and JKD6159_AraC*r.* Data shown are mean CFU and SEM. Note, *** p < 0.001, * p < 0.05.

**Figure 6:**
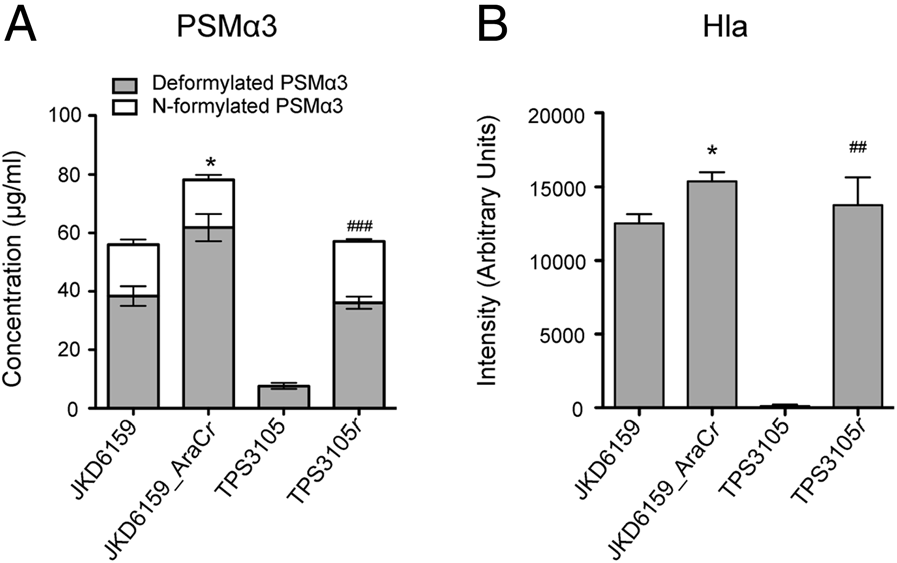
***In vitro *PSM**α**3 and Hla expression of mutant *S. aureus *isolates.** JKD6159 compared with JKD6159_AraC*r.* TPS3105 compared with TPS3105*r*. **(A)** PSMα3 expression measured by HPLC. JKD6159_AraC*r* expressed more PSMα3 than JKD6159 (p = 0.0325). TPS3105*r* expressed more PSMα3 than TPS3105 (p < 0.0001). Data shown are mean concentration (μg/ml), presented as vertical stacked bars and SEM. Deformylated PSMα3 is shown in grey bars. N-formylated PSMα3 is shown in white bars. **(B)** Hla expression measured by quantitative Western blot. There was a small but statistically significant increase in Hla production by JKD6159_AraC*r* (p = 0.0473). TPS3105*r* expressed more Hla than TPS3105 (p = 0.0019) Data shown are mean intensity of bands in arbitrary units and SEM. Note, *p < 0.05, compared to JKD6159. Note also, ^###^p < 0.001 and ^##^p < 0.01, compared to TPS3105.

TPS3105 contained a frame-shift mutation within *agrA* (Sa_JKD6159 nucleotide 2096502) and a further substitution (G to A) within *agrA* at nucleotide 2096569), while TPS3106 contained an ~356 bp deletion spanning the *agr* effector molecule, RNAIII (deletion spanning nucleotides 2093372 to 2093728). These mutations suggested these isolates were *agr* deficient. To demonstrate that these changes explained the reduced exotoxin expression and loss of virulence in TPS3105 we repaired *agrA* using allelic exchange to create TPS3105*r* (the parental strain TPS3105 now containing an intact *agrA*)*.* Quantitative real time RT-PCR for *RNAIII* demonstrated that TPS3105*r* produced 325-fold more *RNAIII* than TPS3105. Virulence was also restored and TPS3105*r* caused greater weight loss, skin lesion area and CFU recovery from lesions compared to the parental strain TPS3105 (p < 0.0001, Figure 5). There was no significant difference between JKD6159 and TPS3105*r* in all outcome measures in the mouse skin infection model (Figure 5). These experiments show that intact *agr* is essential for the virulence of ST93 CA-MRSA. The *agrA* repaired mutant of TPS3105, TPS3105*r* expressed significantly greater amounts of PSMα3 (p < 0.0001) and Hla (p = 0.0019), consistent with *agr* control of these virulence determinants (Figure 6). Thus, despite the genetic divergence of ST93 from other *S. aureus*[14], the molecular foundation of virulence for this CA-MRSA clone is similar in this respect to USA300 [9,26,27] and other *S. aureus* strains [28,29], where the importance of *agr* has been very well established.

### The AraC/XylS regulator (AryK) enhanced Hla expression and virulence in ST93 CA-MRSA

The SNP at position 92551 in SAA6159_00084 introduced a premature stop codon and created a pseudogene within SAA6159_00084 in JDK6159, however the gene was intact in TPS3106. The intact version of this gene, which was also intact in 19 other publically available *S. aureus* genome sequences we examined, encodes a previously uncharacterized AraC/XylS family regulatory protein. While the virulence attenuation in TPS3106 was likely a direct result of the *agr* deficiency, we also wanted to determine if the novel regulator mutation in SAA6159_00084 impacted the virulence in ST93 *S. aureus*.

To test the hypothesis that SAA6159_00084 encoded a regulator of virulence, we repaired the premature stop codon in SAA6159_00084 in JKD6159 using allelic exchange to generate strain JKD6159_AraC*r.* To confirm we had not introduced additional DNA changes during allelic exchange we sequenced the whole genome of JKD6159_AraC*r* and found no additional mutations (35× coverage). JKD6159_AraC*r* encoding an intact copy of SAA6159_00084 demonstrated a modest, but significant increase in virulence as indicated by lesion size (p < 0.0001) and weight loss in the mouse skin infection assay (p = 0.0311, Figure 5), suggesting that this protein is a positive regulator of virulence in CA-MRSA strains. JKD6159_AraC*r* expressed more PSMα3 (p = 0.0325) and Hla (p = 0.0473) than its parental strain JKD6159 that was consistent with an increase mouse skin lesion size (Figure 6). We propose the name *aryK* for SAA6159_00084 (**Ar**aC famil**y**-li**k**e gene).

### RNAseq demonstrates global regulatory impact of AryK

**Figure 7:**
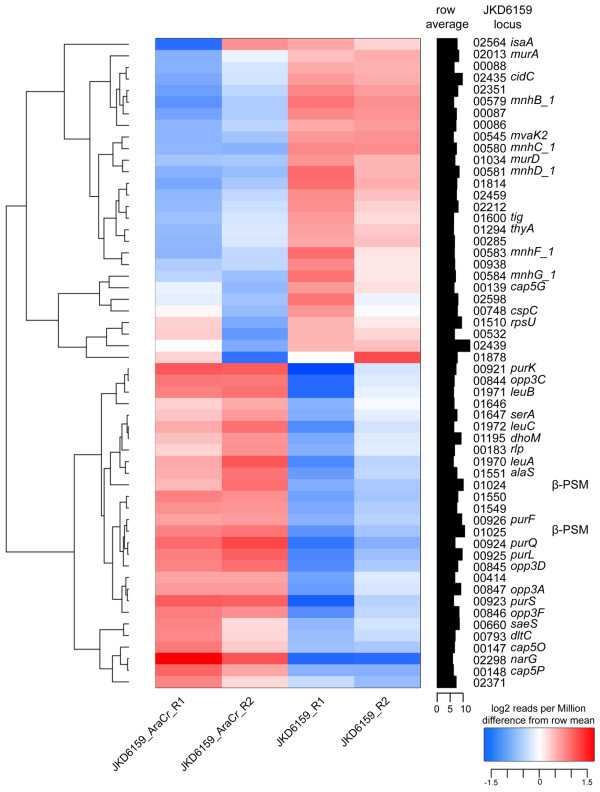
**Heatmap of RNA Sequencing comparing JKD6159 (*aryK *inactive) to JKD6159_AraC*r *(*aryK *intact).** RNA seq was performed in duplicate from stationary phase cultures. This heatmap, clustered on expression profiles, was created based on log_2_ transformed counts to identify consistent changes in expression profiles between strains. To be included in the heat map, genes were required to have at least 1000 counts (reads), totaled over all samples, where the standard deviation of log_2_ expression differences had to exceed two. The heatmap highlights significant *aryK*-dependent changes, in particular genes involved in the regulation of central metabolic functions.

To investigate the regulatory impact of AryK, RNAseq was performed using RNA extracted from stationary phase cultures (Figure 7). This growth phase was selected as we reasoned that AryK might be interacting with *agr* and thus any impacts on Hla expression would be greatest at this time. A small number of virulence-associated loci were down regulated in the *aryK* mutant (JKD6159), including beta-type phenol soluble modulins (SAA6159_01024 and SAA6159_01025), and the virulence regulator *saeS*. However, the most dramatic and significant transcriptional changes were found in genes involved in central metabolic functions. Using the Kyoto Encyclopedia of Genes and Genomes (KEGG) pathway analysis ( http://www.genome.jp) [30] the major pathways where differential transcription occurred were down regulation of genes linked to purine metabolism (*purK, purS, purQ, purL, purF*), valine, leucine and isoleucine biosynthesis (*leuA*, *leuB*, *leuC*), and oligopeptide transport (*opp3C, opp3D, opp3F, opp3A*). In contrast up regulation of genes encoding cation transport systems (*mnhB_1, mnhC_1, mnhD_1, mnhF_1, mnhG_1*) was found.

Here, we have clearly demonstrated that *agr* is the major "on-off" switch for virulence in ST93 CA-MRSA, but we also found that other genetic changes are impacting virulence gene regulation in a clone-specific manner. We speculate that the inactivation of *aryK* may have been an evolutionary response by ST93 CA-MRSA to modulate or fine-tune the amount of Hla and other factors required for host persistence. There are six AraC/XylS family regulators in *S. aureus* (SA0097, SA0215, SA0622, SA1337, SA2092, SA2169; *S. aureus* strain N315 locus tags). Two of these, Rbf (SA0622) and Rsp (SA2169) have been studied and demonstrated in other *S. aureus* strains to regulate biofilm formation and modulate expression of surface-associated proteins [24,25,31]. In contrast, we found that *aryK* increases Hla expression and virulence, acting as a positive regulator of virulence by directly or indirectly upregulating exotoxin expression, without an apparent effect on *agr* expression in stationary phase.

## Conclusions

In this study, we have obtained insights into the genetic basis for the increased virulence of ST93 by using a combination of comparative and functional genomics. We have demonstrated the key role of Hla and *agr* and shown how an additional novel regulatory gene, *aryK* by a loss-of-function point mutation, is modulating virulence in this clone. Quantification of exotoxin expression in a larger collection of ST93 strains demonstrated that the findings in strain JKD6159 are relevant to the majority of the ST93 population isolated from around Australia as exotoxin expression in JKD6159 is representative of most of the ST93 population. Our study highlights the power of comparative genomics to uncover new regulators of virulence but it also shows the complex nature of these changes even in closely related bacterial populations. Careful strain selection, detailed comparative genomics analyses, and functional genomic studies by creating multiple genetic changes in one strain will be required to gain a full insight into the genetic basis for the emergence and hypervirulence of ST93 CA-MRSA.

## Methods

### Ethics statement

This study was performed in accordance with the Australian Prevention of Cruelty to Animals Act 1986 and the Australian code of practice for the care and use of animals for scientific purposes. The protocol was approved by the Animal Ethics Committee of the University of Melbourne (Permit Number: 0911248.2).

### Bacterial strains and culture

Bacterial strains used in this study are summarized in Table 1 (international clone collection) and Additional file 1 (ST93 strain collection), and include the ST93 reference strains JKD6159, USA300 strain FPR3757 [19], ST30 strain JKD6177, and the HA-MRSA ST239 clone JDK6009 [20], as well as 58 additional ST93 collected from around Australia and previously reported [17]. For all experiments except exotoxin expression bacteria were grown in brain heart infusion broth (BHI, Oxoid). For the mouse skin infection assay, *S. aureus* were harvested at the stationary phase of growth after 18 hours incubation (OD_600_ ~ 2.0), washed, diluted and resuspended in PBS. The bacterial inoculum (CFU) and viable counts were determined by plating onto BHI agar and colony enumeration.

For LukF-PV expression experiments, bacteria were grown in CCY media (3% yeast extract (Oxoid), 2% Bacto Casamino Acids (Difco), 2.3% sodium pyruvate (Sigma-Aldrich), 0.63% Na_2_HPO_4_, 0.041% KH_2_PO_4_, pH 6.7). For α-hemolysin (Hla) and PSMα3 expression experiments, bacteria were grown in tryptone soy broth (TSB, Oxoid). Overnight cultures were diluted 1:100 into fresh media and then incubated at 37°C with shaking (180 rpm) until stationary phase was achieved. For LukF-PV detection, isolates were cultured for 8 hours (OD_600_ ~ 1.8); for Hla detection, isolates were cultured for approximately 3 hours (OD_600_ ~ 1.8); and for PSMα3 detection, isolates were cultured for 24 hours (OD_600_ ~ 2.0). Culture supernatants were harvested by centrifugation and filter sterilized. These were performed in at least triplicate for each *S. aureus* strain.

### Detection of LukF-PV and Hla by western blotting

Trichloroacetic acid was added to culture supernatants and incubated at 4°C overnight. Precipitates were then harvested by centrifugation, washed with acetone, air-dried and solubilized in a SDS and 2-mercaptoethanol containing sample buffer. The proteins were separated on 12% SDS-PAGE. A peptide sequence specific to LukF-PV, HWIGNNYKDENRATHT was synthesized and HRP conjugated polyclonal chicken IgY was raised against this peptide (Genscript). This antibody was used to detect LukF-PV with enhanced chemiluminescence. Images generated from the western blots were quantitated using GS800 Calibrated Densitometer (BioRad) and Image J [32].

Hla was detected using a polyclonal rabbit anti-Hla (Sigma-Aldrich), in buffer containing 20 mM DEPC to inhibit non-specific protein A binding and HRP conjugated sheep anti-rabbit secondary antibody (Millipore) with enhanced chemiluminescence detection [33]. For comparison of JKD6159 versus international clone collection (Table 1), images generated from the Hla western blots were quantitated using GS800 Calibrated Densitometer (BioRad) and Image J [32].

Subsequently, for comparison of JKD6159 and other ST93 strains (Table 1), detection of chemiluminescence was performed using the MF-ChemiBIS 3.2 platform (DNR Bioimaging systems). Quantitation was performed using Image J [32].

### Detection of PSMα3 expression

HPLC chromatography was performed on an Agilent Technology 1200 Series system with an analytical Agilent Eclipse XDB-C18 (4.6 mm × 150 mm) column. A water/acetonitrile gradient (0.1% trifluoroacetic acid) from 0 – 100% acetonitrile over 28 min at a flow rate of 1 mL/min was used. The total run time was 32 min, and peaks were quantified at a wavelength of 214 nm. The deformylated and formylated form of PSMα3 MEFVAKLFKFFKDLLGKFLGNN was identified in the *S. aureus* TSB culture supernatants by comparison of their retention times to a commercially synthesized PSMα3 standard (GenScript) and by spiking the samples with the synthesized standards. The identity of the deformylated peptide present in the samples was confirmed by analysing collected fractions by ESI-MS. There was only one peptide present in this fraction; the deformylated form of PSMα3. In contrast, other peptides were observed in the fractions of USA300, JKD6272, TPS3104, TPS3105*r*, and JKD6159_AraC*r* containing the N-formylated form of PSMα3. In these cases, the percentage of N-formylated PSMα3 peptide was determined using the total ion count of the major peaks in the ESI-MS and the peak area of the HPLC chromatogram was adjusted accordingly. The concentrations of the deformylated and formylated forms of PSMα3 were determined by comparison of their peak areas to those of the synthesized standards. The standard curves were constructed in the concentration range of 6.2 – 100 μg/ mL and were linear over this range.

### DNA methods, molecular techniques and construction of mutants

DNA was extracted using the GenElute kit according to the manufacturer’s instructions (Sigma-Aldrich). A *lukSF-PV* knockout, *hla* knockout and a repaired *agrA* of TPS3105 were generated according to the published method [34]. For the knockouts, flanking sequences were amplified and ligated prior to cloning with pKOR1. For allelic replacement to generate TPS3105*r,* a PCR product of *agrA* from JKD6159 was cloned with pKOR1. For allelic replacement JKD6159_AraC*r,* a PCR product of this AraC regulator from TPS3106 was cloned with pKOR1. The deletion of the whole *psmα* locus in JKD6159, chromosomal restoration of *psmα* in JKD6159∆*psmα* and the restoration of Hla expression in JKD6159∆*hla* were conducted using the pIMAY protocol described by Monk et al. [35]. Knockout and restoration amplimers were cloned into pIMAY by SLIC [36]. The primers used are listed in Additional file 11. The knockout and restoration clones were confirmed by PCR and Sanger sequencing of the mutated locus. Mutations were further validated with functional assays of activity, which included sheep blood hemolysis, western blot, and/or HPLC.

### Mouse skin infection assay

Mice were infected with *S. aureus* as previously described [14]. Briefly, six-week-old female BALB/c mice were infected by intradermal injection with 10^8^ CFU of *S. aureus.* Mice were assessed and weighed daily for five days. Mice were culled on the 5th day and lesion size measured and CFU recovered from infected tissues by homogenization and colony enumeration on BHI. For each *S. aureus* strain, at least 10 mice were assessed.

### Genome sequencing

Genome sequences for three ST93 strains (TPS3104, TPS3105, TPS3106) were obtained from an Illumina GAIIx analyzer using 100 bp paired-end chemistry with a mean fold coverage of 331×. Genome sequencing of the two laboratory-induced mutants JKD6159∆*hla* (TPS3265) and JKD6159_AraC*r* (TPS3268) was performed using Ion Torrent sequencing technology.

### Comparative genomics

A read mapping approach was used to compare the sequences from all isolates used in this study, as previously described [14,37]. Briefly, the reads from all genomes were aligned to the JKD6159 reference using SHRiMP 2.0 [38]. SNPs were identified using Nesoni v0.60 [ http://www.bioinformatics.net.au]. Using the whole genome sequence of JKD6159 as a reference, a global SNP analysis was performed, and allelic variability at any nucleotide position was tallied to generate a global SNP analysis for every genome compared to JKD6159.

### Quantitative RT-PCR for RNAIII expression

To investigate activity of the *agr* locus (RNAIII) qRT-PCR was performed for RNAIII as previously described [37]. Briefly, RNA was prepared as previously described with two on-column DNase I digestion steps and cDNA synthesis using SuperScript II reverse transcriptase (Invitrogen). Relative expression was determined as previously described and was normalised against *gyrB*. Results were obtained from 3 biological replicates each performed in triplicate.

### RNA sequencing

*Staphylococcus aureus* strains JKD6159 and JKD6159_AraC*r* were grown to early stationary culture as described above. For RNA protection, 0.5 volumes of RNAlater® RNA stabilization reagent (Qiagen) was added immediately to the liquid culture and allowed to incubate with the bacterial suspension for 15 minutes at room temperature. Cells were pelleted at 5,000 × g for 5 minutes followed by RNA extraction using RNeasy mini kit (Qiagen) and two rounds of DNase I digestion (Qiagen) according to the manufacturer’s instruction. RNA concentration was quantified using Qubit® 2.0 Fluorometer and RNA quality assessed using Agilent 2100 Bioanalyzer. Ten μg of total RNA from the stationary growth phase with RNA intergrity number (RIN) greater than 7 was used in RNA-seq. Ribosomal depletion, cDNA library preparation and pair ended sequencing using HiSeq2000 sequencing platform was performed by Beijing Genome Institute (Hong Kong, China). RNAseq was performed on two biological samples for each strain.

RNAseq reads were mapped onto the JKD6159 reference genome [23], using SHRiMP 2.2.2 [39]. Alignment to CDS features from each biological replicate of each strain provided counts that were a measure of mRNA levels. Counts were normalized using the trimmed-mean normalization function in edgeR, part of the BioConductor package [40]. A heat map was created based on log_2_ transformed counts to identify consistent changes in expression profiles between strains. To be included in the heat map, genes were required to have at least 1000 counts, totaled over all samples, where and the standard deviation of the log_2_ expression levels had to exceed two.

### Statistical analysis

Percentage mouse weight change at day 5, viable counts of *S. aureus* in mouse tissues and skin lesion area of each isolate, Hla, LukF-PV and PSMα3 expression versus JKD6159 were analyzed using an unpaired t test. A similar analysis was used to analyze virulence outcome measures and exotoxin expression between TPS3105 and TPS3105*r*. (There was no difference in results when Bonferonni analysis was performed). All analyses were performed using Prism 5 for Macintosh v5.0b (GraphPad Software Inc.).

### Availability of supporting data

The data sets supporting the results of this article are in the NCBI Sequence Read Archive under study accession SRP004474.2 and the NCBI BioProject Archive under study accession PRJNA217697.

## Competing interest

No author has any competing interests to declare.

## Authors’ contributions

Conceived the project, TPS, BPH, KYLC, JKD; performed the experiments, KYLC, IRM, YHL, JLP, GWC, JS, KLT; analysed the data, KYLC, YHL, TPS, BPH, TS, KLT; wrote the manuscript, KYLC, BPH, TPS. All authors read and approved the final manuscript.

## Authors’ information

Timothy P. Stinear and Benjamin P. Howden are the Joint Senior Authors.

## Supplementary Material

Additional file 1 ***Staphylococcus aureus *** **ST93 strains used in this study.** Click here for file

Additional file 2 **Expression of PSMα3 by ST93 strains and USA300.** (A) Expression of deformylated PSMα3. (B) Expression of N-formylated PSMα3. Data shown are mean concentration (μg/ml) and SEM. Click here for file

Additional file 3 **Expression of Hla by ST93 strains and USA300.** Hla expression measured by quantitative Western blot. Data shown are mean intensity of bands in arbitrary units and SEM. Click here for file

Additional file 4 **Hla Western Blot of JKD6159, JKD6159∆** ***hla* and JKD6159∆** ***hla r *** **(A) Western Blot demonstrating that JKD6159∆** ***hla *** **does not express Hla by Western Blot and that complementation of this mutant (JKD6159∆** ***hla r*) results in restoration of Hla expression.** (B) Arrangement of PCR primers used PCR screen of JKD6159∆*hla* and JKD6159∆*hla r.* (C) PCR screen of 25 randomly selected *S. aureus* colonies obtained from two mice (mouse 4 and mouse 7) post skin infection with JKD6159∆*hla r.* The PCR primers used flank the region deleted in *hla* for the mutant and show incomplete penetration of the bacterial population with the repaired version of *hla* (17/25 with an intact allele for mouse 4 and 21/25 for mouse 7), thereby explaining the inability of the repaired mutant to fully restore the virulence phenotype in this infection model. Click here for file

Additional file 5 **Detection of formylated PSMα3 in JKD6159, JKD6159∆** ***psmα* and JKD6159∆** ***psmα r *** **by HPLC of culture filtrates.** JKD6159∆*psmα* did not produce formylated PSMα3. Complementation of this strain resulted in restoration of formylated PSMα3 expression. In all strains δ-toxin expression was maintained. Click here for file

Additional file 6 **LukF-PV Western Blot of JKD6159 and JKD6159∆** ***lukSF-PV.*** Western Blot demonstrating that JKD6159∆*lukSF-PV* does not express LukF-PV. Click here for file

Additional file 7 **Table of *de novo *** **assembly characteristics for *S. aureus* strains TPS3104, TPS3105 and TPS3106.** Click here for file

Additional file 8 Table of single nucleotide differences between JKD6159 and TPS3104. Click here for file

Additional file 9 Table of single nucleotide differences between JKD6159 and TPS3105. Click here for file

Additional file 10 Table of single nucleotide differences between JKD6159 and TPS3106. Click here for file

Additional file 11 Table of primers used in this study. Click here for file

## References

[B1] David MZ Daum RS Community-associated methicillin-resistant *Staphylococcus aureus*: epidemiology and clinical consequences of an emerging epidemic Clin Microbiol Rev 2010 23 3 616 687 10.1128/CMR.00081-09 20610826 PMC2901661

[B2] Loffler B Hussain M Grundmeier M Bruck M Holzinger D Varga G Roth J Kahl BC Proctor RA Peters G *Staphylococcus aureus* Panton-Valentine leukocidin is a very potent cytotoxic factor for human neutrophils PLoS Pathog 2010 6 1 e1000715 10.1371/journal.ppat.1000715 20072612 PMC2798753

[B3] Diep BA Chan L Tattevin P Kajikawa O Martin TR Basuino L Mai TT Marbach H Braughton KR Whitney AR Polymorphonuclear leukocytes mediate *Staphylococcus aureus* Panton-Valentine leukocidin-induced lung inflammation and injury Proc Natl Acad Sci U S A 2010 107 12 5587 5592 10.1073/pnas.0912403107 20231457 PMC2851770

[B4] Kobayashi SD Malachowa N Whitney AR Braughton KR Gardner DJ Long D Bubeck Wardenburg J Schneewind O Otto M Deleo FR Comparative analysis of USA300 virulence determinants in a rabbit model of skin and soft tissue infection J Infect Dis 2011 204 6 937 941 10.1093/infdis/jir441 21849291 PMC3156927

[B5] Lipinska U Hermans K Meulemans L Dumitrescu O Badiou C Duchateau L Haesebrouck F Etienne J Lina G Panton-Valentine leukocidin does play a role in the early stage of *Staphylococcus aureus* skin infections: a rabbit model PLoS One 2011 6 8 e22864 10.1371/journal.pone.0022864 21850240 PMC3151264

[B6] Li M Diep BA Villaruz AE Braughton KR Jiang X DeLeo FR Chambers HF Lu Y Otto M Evolution of virulence in epidemic community-associated methicillin-resistant *Staphylococcus aureus* Proc Natl Acad Sci U S A 2009 106 14 5883 5888 10.1073/pnas.0900743106 19293374 PMC2667066

[B7] Li M Cheung GY Hu J Wang D Joo HS Deleo FR Otto M Comparative analysis of virulence and toxin expression of global community-associated methicillin-resistant *Staphylococcus aureus* strains J Infect Dis 2010 202 12 1866 1876 10.1086/657419 21050125 PMC3058913

[B8] Bhakdi S Tranum-Jensen J Alpha-toxin of *Staphylococcus aureus* Microbiol Rev 1991 55 4 733 751 1779933 10.1128/mr.55.4.733-751.1991 PMC372845

[B9] Bubeck Wardenburg J Bae T Otto M Deleo FR Schneewind O Poring over pores: alpha-hemolysin and Panton-Valentine leukocidin in *Staphylococcus aureus* pneumonia Nat Med 2007 13 12 1405 1406 10.1038/nm1207-1405 18064027

[B10] Kennedy AD Bubeck Wardenburg J Gardner DJ Long D Whitney AR Braughton KR Schneewind O DeLeo FR Targeting of alpha-hemolysin by active or passive immunization decreases severity of USA300 skin infection in a mouse model J Infect Dis 2010 202 7 1050 1058 10.1086/656043 20726702 PMC2945289

[B11] Wang R Braughton KR Kretschmer D Bach TH Queck SY Li M Kennedy AD Dorward DW Klebanoff SJ Peschel A Identification of novel cytolytic peptides as key virulence determinants for community-associated MRSA Nat Med 2007 13 12 1510 1514 10.1038/nm1656 17994102

[B12] Cheung GY Duong AC Otto M Direct and synergistic hemolysis caused by *Staphylococcus* phenol-soluble modulins: implications for diagnosis and pathogenesis Microbes Infect 2012 14 4 380 386 10.1016/j.micinf.2011.11.013 22178792 PMC3299937

[B13] Coombs GW Nimmo GR Pearson JC Christiansen KJ Bell JM Collignon PJ McLaws ML Resistance AGfA Prevalence of MRSA strains among *Staphylococcus aureus* isolated from outpatients, 2006 Commun Dis Intell 2009 33 1 10 20 10.33321/cdi.2009.33.2 19618763

[B14] Chua KY Seemann T Harrison PF Monagle S Korman TM Johnson PD Coombs GW Howden BO Davies JK Howden BP The dominant Australian community-acquired methicillin-resistant *Staphylococcus aureus* clone ST93-IV [2B] is highly virulent and genetically distinct PLoS One 2011 6 10 e25887 10.1371/journal.pone.0025887 21991381 PMC3185049

[B15] Tong SY Sharma-Kuinkel BK Thaden JT Whitney AR Yang SJ Mishra NN Rude T Lilliebridge RA Selim MA Ahn SH Virulence of endemic nonpigmented northern Australian *Staphylococcus aureus* clone (clonal complex 75, *S. argenteus*) is not augmented by staphyloxanthin J Infect Dis 2013 208 3 520 527 10.1093/infdis/jit173 23599317 PMC3699000

[B16] Labandeira-Rey M Couzon F Boisset S Brown EL Bes M Benito Y Barbu EM Vazquez V Hook M Etienne J *Staphylococcus aureus* Panton-Valentine leukocidin causes necrotizing pneumonia Science 2007 315 5815 1130 1133 10.1126/science.1137165 17234914

[B17] Coombs GW Goering RV Chua KY Monecke S Howden BP Stinear TP Ehricht R O’Brien FG Christiansen KJ The molecular epidemiology of the highly virulent ST93 Australian community *Staphylococcus aureus* strain PLoS One 2012 7 8 e43037 10.1371/journal.pone.0043037 22900085 PMC3416834

[B18] Somerville GA Cockayne A Durr M Peschel A Otto M Musser JM Synthesis and deformylation of *Staphylococcus aureus* delta-toxin are linked to tricarboxylic acid cycle activity J Bacteriol 2003 185 22 6686 6694 10.1128/JB.185.22.6686-6694.2003 14594843 PMC262117

[B19] Diep BA Gill SR Chang RF Phan TH Chen JH Davidson MG Lin F Lin J Carleton HA Mongodin EF Complete genome sequence of USA300, an epidemic clone of community-acquired meticillin-resistant *Staphylococcus aureus* Lancet 2006 367 9512 731 739 10.1016/S0140-6736(06)68231-7 16517273

[B20] Howden BP Stinear TP Allen DL Johnson PD Ward PB Davies JK Genomic analysis reveals a point mutation in the two-component sensor gene *graS* that leads to intermediate vancomycin resistance in clinical *Staphylococcus aureus* Antimicrob Agents Chemother 2008 52 10 3755 3762 10.1128/AAC.01613-07 18644967 PMC2565880

[B21] Voyich JM Otto M Mathema B Braughton KR Whitney AR Welty D Long RD Dorward DW Gardner DJ Lina G Is Panton-Valentine leukocidin the major virulence determinant in community-associated methicillin-resistant *Staphylococcus aureus* disease? J Infect Dis 2006 194 12 1761 1770 10.1086/509506 17109350

[B22] Bubeck Wardenburg J Palazzolo-Ballance AM Otto M Schneewind O DeLeo FR Panton-Valentine leukocidin is not a virulence determinant in murine models of community-associated methicillin-resistant *Staphylococcus aureus* disease J Infect Dis 2008 198 8 1166 1170 10.1086/592053 18729780 PMC2574921

[B23] Chua K Seemann T Harrison PF Davies JK Coutts SJ Chen H Haring V Moore R Howden BP Stinear TP Complete genome sequence of *Staphylococcus aureus* strain JKD6159, a unique Australian clone of ST93-IV community methicillin-resistant *Staphylococcus aureus* J Bacteriol 2010 192 20 5556 5557 10.1128/JB.00878-10 20729356 PMC2950503

[B24] Cue D Lei MG Luong TT Kuechenmeister L Dunman PM O’Donnell S Rowe S O’Gara JP Lee CY Rbf promotes biofilm formation by *Staphylococcus aureus* via repression of *icaR*, a negative regulator of *icaADBC* J Bacteriol 2009 191 20 6363 6373 10.1128/JB.00913-09 19684134 PMC2753044

[B25] Lei MG Cue D Roux CM Dunman PM Lee CY Rsp inhibits attachment and biofilm formation by repressing *fnbA* in *Staphylococcus aureus* MW2 J Bacteriol 2011 193 19 5231 5241 10.1128/JB.05454-11 21804010 PMC3187379

[B26] Montgomery CP Boyle-Vavra S Daum RS Importance of the global regulators *agr* and *saeRS* in the pathogenesis of CA-MRSA USA300 infection PLoS One 2010 5 12 e15177 10.1371/journal.pone.0015177 21151999 PMC2996312

[B27] Cheung GY Wang R Khan BA Sturdevant DE Otto M Role of the accessory gene regulator *agr i*n community-associated methicillin-resistant *Staphylococcus aureus* pathogenesis Infect Immun 2011 79 5 1927 1935 10.1128/IAI.00046-11 21402769 PMC3088142

[B28] Cheung AL Eberhardt KJ Chung E Yeaman MR Sullam PM Ramos M Bayer AS Diminished virulence of a *sar-/agr-* mutant of *Staphylococcus aureus* in the rabbit model of endocarditis J Clin Invest 1994 94 5 1815 1822 10.1172/JCI117530 7962526 PMC294579

[B29] Wright JS 3rd Jin R Novick RP Transient interference with staphylococcal quorum sensing blocks abscess formation Proc Natl Acad Sci U S A 2005 102 5 1691 1696 10.1073/pnas.0407661102 15665088 PMC547845

[B30] Kanehisa M Goto S Sato Y Furumichi M Tanabe M KEGG for integration and interpretation of large-scale molecular data sets Nucleic Acids Res 2012 40 Database issue D109 D114 22080510 10.1093/nar/gkr988 PMC3245020

[B31] Lim Y Jana M Luong TT Lee CY Control of glucose- and NaCl-induced biofilm formation by *rbf* in *Staphylococcus aureus* J Bacteriol 2004 186 3 722 729 10.1128/JB.186.3.722-729.2004 14729698 PMC321492

[B32] Rasband WS ImageJ Bethesda, Maryland, USA: U S National Institutes of Health available at http:/imagej.nih.gov/ij/, accessed 9 December 2009 1997–2011

[B33] Nguyen HM Rocha MA Chintalacharuvu KR Beenhouwer DO Detection and quantification of Panton-Valentine leukocidin in *Staphylococcus aureus* cultures by ELISA and Western blotting: diethylpyrocarbonate inhibits binding of protein A to IgG J Immunol Methods 2010 356 1–2 1 5 20303971 10.1016/j.jim.2010.03.005 PMC2878937

[B34] Bae T Schneewind O Allelic replacement in *Staphylococcus aureus* with inducible counter-selection Plasmid 2006 55 1 58 63 10.1016/j.plasmid.2005.05.005 16051359

[B35] Monk IR Shah IM Xu M Tan MW Foster TJ Transforming the untransformable: application of direct transformation to manipulate genetically *Staphylococcus aureus* and *Staphylococcus epidermidis* MBio 2012 3 2 e00277 00211 22434850 10.1128/mBio.00277-11 PMC3312211

[B36] Li MZ Elledge SJ Harnessing homologous recombination in vitro to generate recombinant DNA via SLIC Nat Methods 2007 4 3 251 256 10.1038/nmeth1010 17293868

[B37] Howden BP McEvoy CR Allen DL Chua K Gao W Harrison PF Bell J Coombs G Bennett-Wood V Porter JL Evolution of multidrug resistance during *Staphylococcus aureus* infection involves mutation of the essential two component regulator WalKR PLoS Pathog 2011 7 11 e1002359 10.1371/journal.ppat.1002359 22102812 PMC3213104

[B38] Rumble SM Lacroute P Dalca AV Fiume M Sidow A Brudno M SHRiMP: accurate mapping of short color-space reads PLoS Comput Biol 2009 5 5 e1000386 10.1371/journal.pcbi.1000386 19461883 PMC2678294

[B39] David M Dzamba M Lister D Ilie L Brudno M SHRiMP2: sensitive yet practical SHort Read Mapping Bioinformatics 2011 27 7 1011 1012 10.1093/bioinformatics/btr046 21278192

[B40] Robinson MD McCarthy DJ Smyth GK edgeR: a Bioconductor package for differential expression analysis of digital gene expression data Bioinformatics 2010 26 1 139 140 10.1093/bioinformatics/btp616 19910308 PMC2796818

