# Supplementary figures and images for: Hyperexpression of α-hemolysin explains enhanced virulence of sequence type 93 community-associated methicillin-resistant Staphylococcus aureus
Source: BMC Microbiol. 2014 Feb 10;14:31. doi: 10.1186/1471-2180-14-31 (PMC3922988; doi:10.1186/1471-2180-14-31)

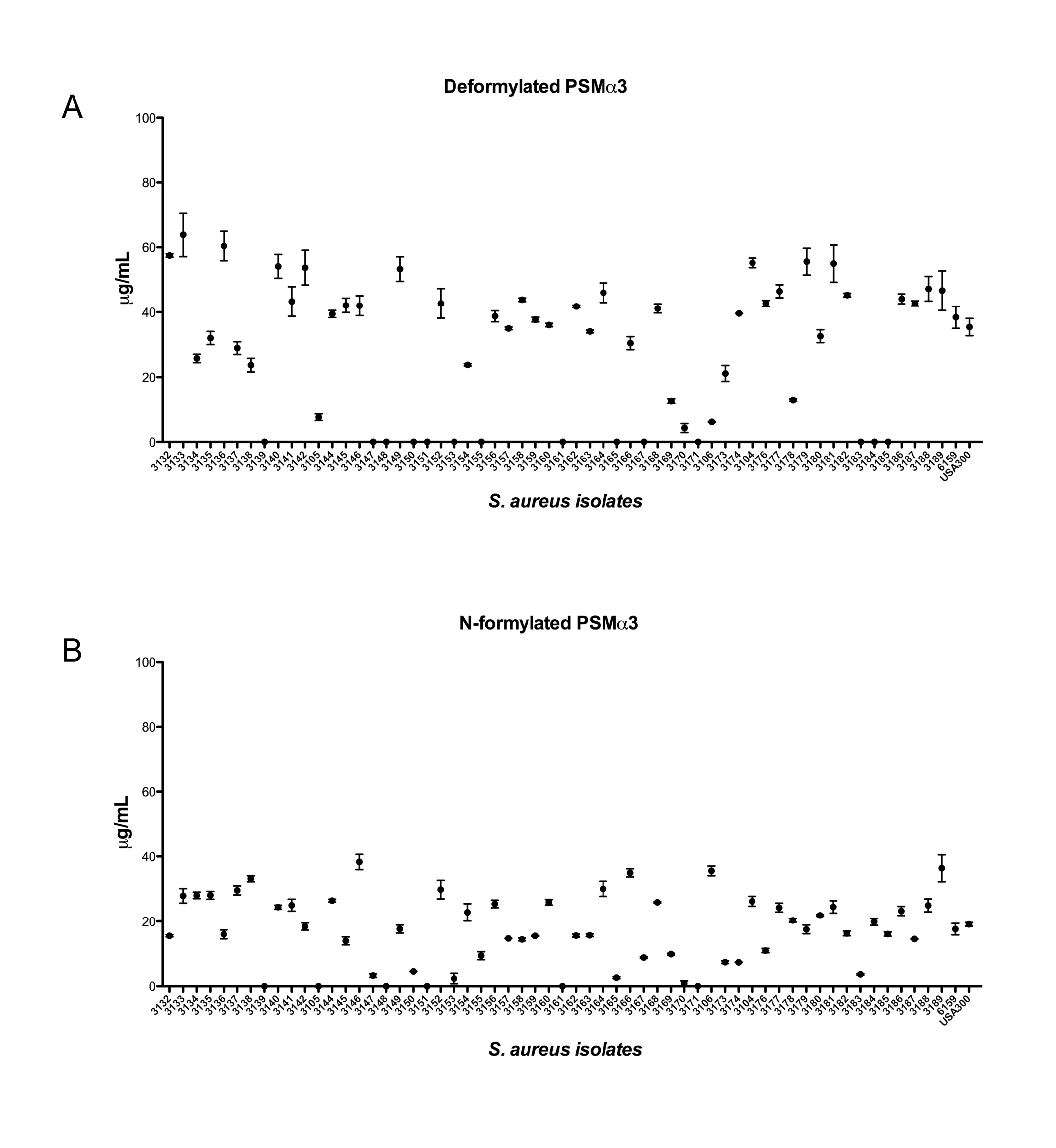

Supplement: Additional file 2 — Expression of PSMα3 by ST93 strains and USA300. (A) Expression of deformylated PSMα3. (B) Expression of N-formylated PSMα3. Data shown are mean concentration (μg/ml) and SEM. [file 1471-2180-14-31-S2.tiff]

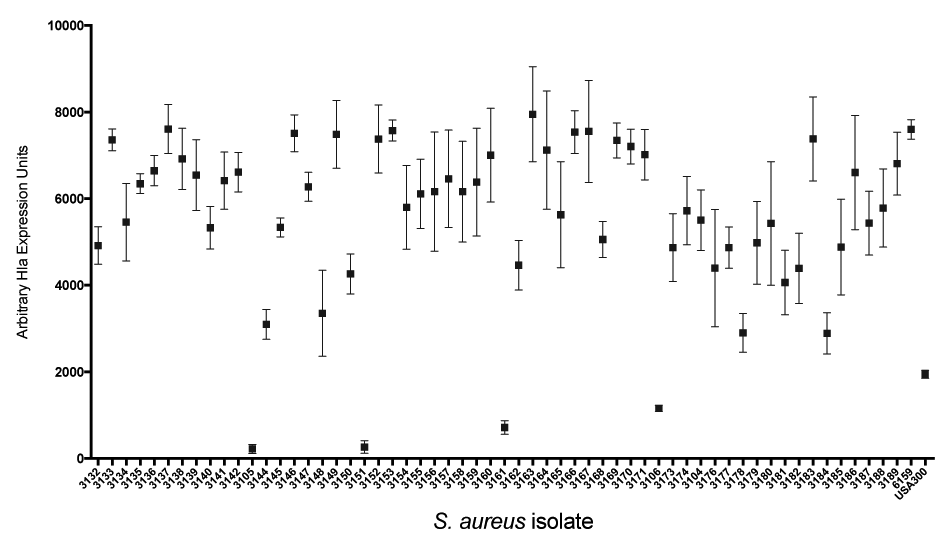

Supplement: Additional file 3 — Expression of Hla by ST93 strains and USA300. Hla expression measured by quantitative Western blot. Data shown are mean intensity of bands in arbitrary units and SEM. [file 1471-2180-14-31-S3.tiff]

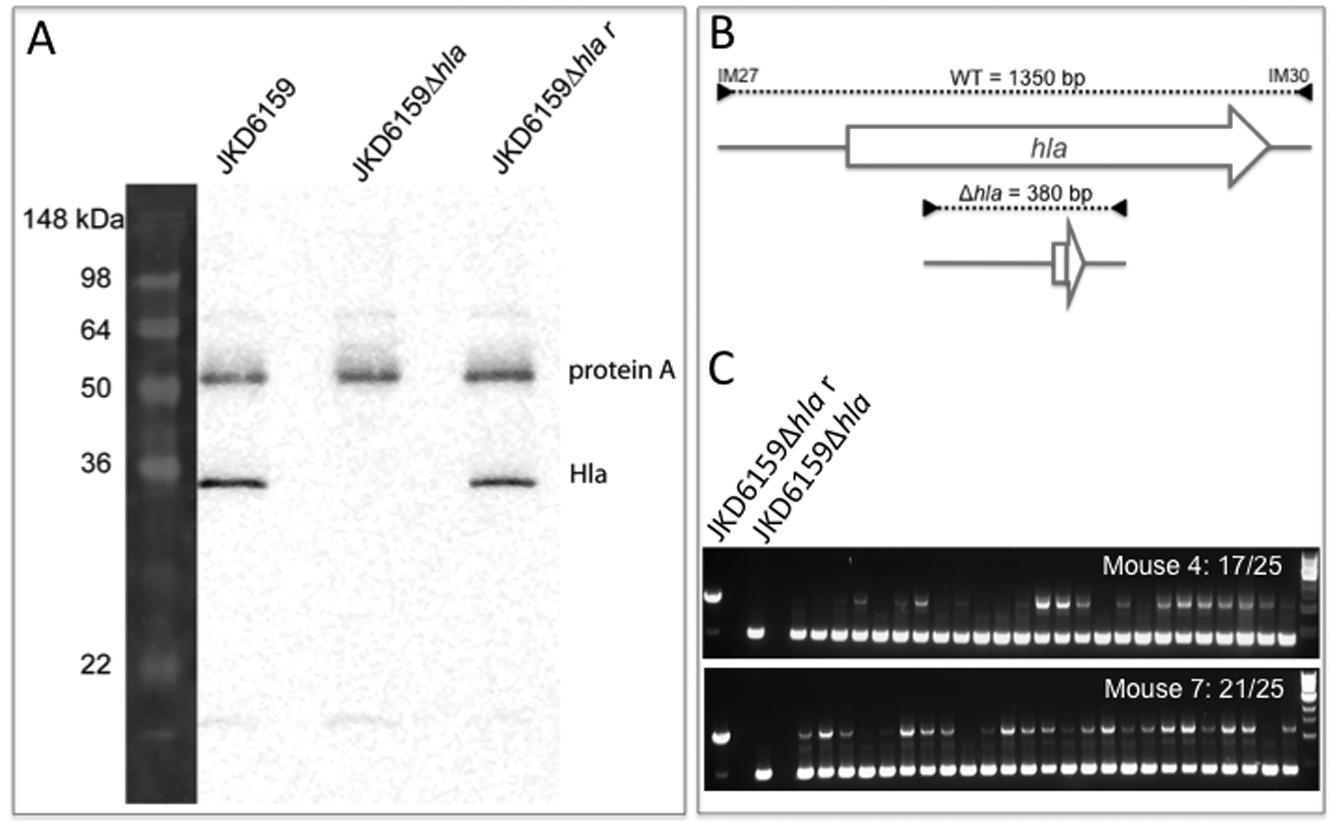

Supplement: Additional file 4 — Hla Western Blot of JKD6159, JKD6159∆ hla and JKD6159∆ hla r (A) Western Blot demonstrating that JKD6159∆ hla does not express Hla by Western Blot and that complementation of this mutant (JKD6159∆ hla r ) results in restoration of Hla expression. (B) Arrangement of PCR primers used PCR screen of JKD6159∆hla and JKD6159∆hla r. (C) PCR screen of 25 randomly selected S. aureus colonies obtained from two mice (mouse 4 and mouse 7) post skin infection with JKD6159∆hla r. The PCR primers used flank the region deleted in hla for the mutant and show incomplete penetration of the bacterial population with the repaired version of hla (17/25 with an intact allele for mouse 4 and 21/25 for mouse 7), thereby explaining the inability of the repaired mutant to fully restore the virulence phenotype in this infection model. [file 1471-2180-14-31-S4.tiff]

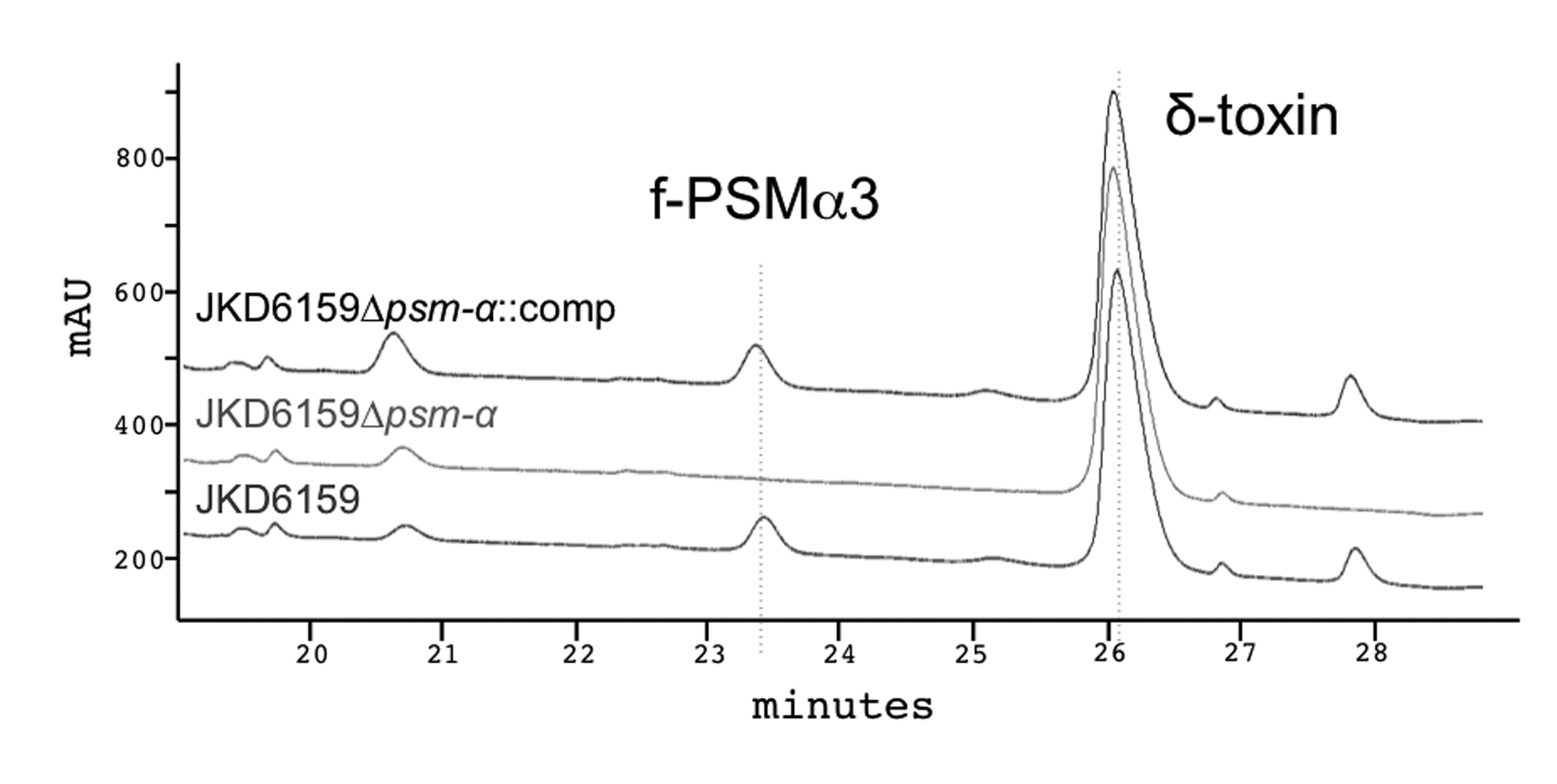

Supplement: Additional file 5 — Detection of formylated PSMα3 in JKD6159, JKD6159∆ psmα and JKD6159∆ psmα r by HPLC of culture filtrates. JKD6159∆psmα did not produce formylated PSMα3. Complementation of this strain resulted in restoration of formylated PSMα3 expression. In all strains δ-toxin expression was maintained. [file 1471-2180-14-31-S5.tiff]

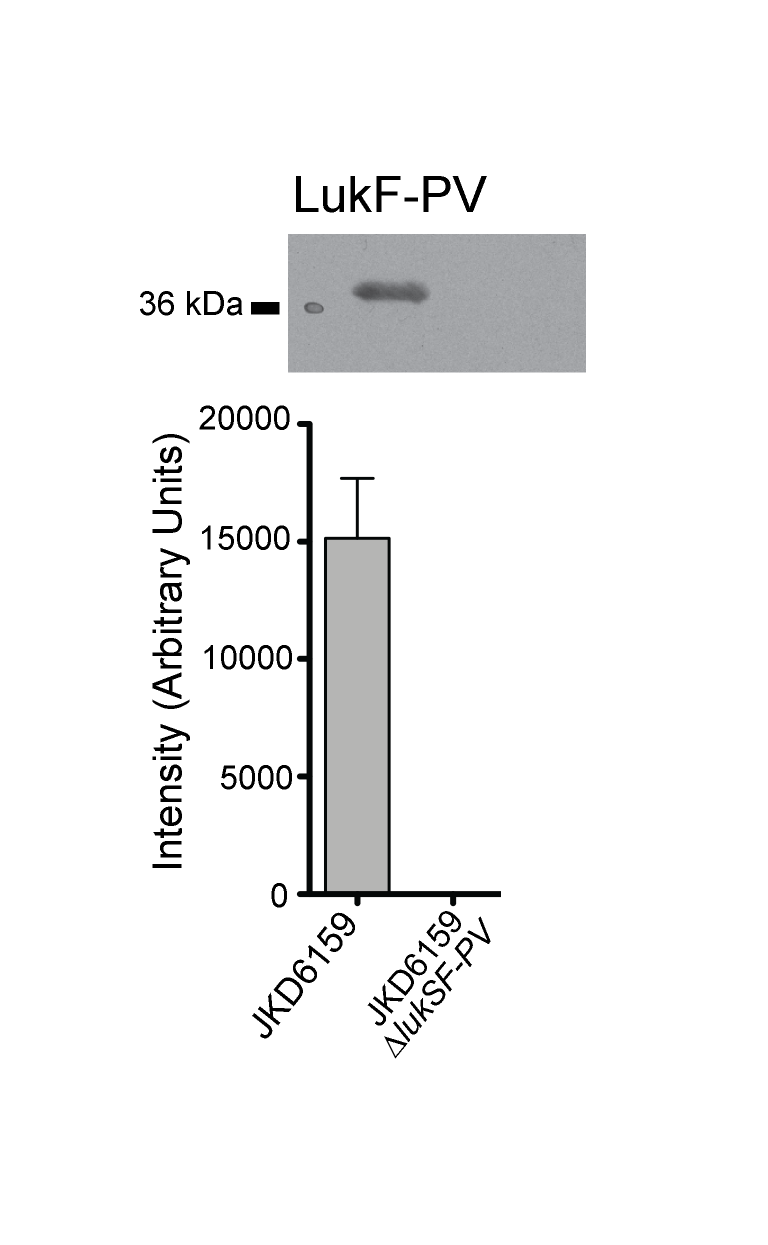

Supplement: Additional file 6 — LukF-PV Western Blot of JKD6159 and JKD6159∆ lukSF-PV. Western Blot demonstrating that JKD6159∆lukSF-PV does not express LukF-PV. [file 1471-2180-14-31-S6.tiff]
