# Supplementary material for: Hyperexpression of α-hemolysin explains enhanced virulence of sequence type 93 community-associated methicillin-resistant Staphylococcus aureus
Source: BMC Microbiol. 2014 Feb 10;14:31. doi: 10.1186/1471-2180-14-31 (PMC3922988; doi:10.1186/1471-2180-14-31)
Supplement: Additional file 7 — Table of de novo assembly characteristics for S. aureus strains TPS3104, TPS3105 and TPS3106. [file 1471-2180-14-31-S7.docx]

**Additional file 7: *de novo* assembly characteristics for *S. aureus* strains TPS3104, TPS3105 and TPS3105.**

| **Strain** | **Genome size** | **Number of Contigs** | **N50** | **Maximum contig length** | **Number of reads** |
| --- | --- | --- | --- | --- | --- |
| TPS3104 | 2,808,814 bp | 701 | 107,063 bp | 299,283 bp | 6,299,900 |
| TPS3105 | 2,746,826 bp | 620 | 146,174 bp | 395,614 bp | 7,946,972 |
| TPS3106 | 2,810,394 bp | 621 | 112,447 bp | 258,500 bp | 8,000,000 |

**Note:** JKD6159 chromosome and plasmid length 2,832,164 bp.
