# Supplementary material for: Hyperexpression of α-hemolysin explains enhanced virulence of sequence type 93 community-associated methicillin-resistant Staphylococcus aureus
Source: BMC Microbiol. 2014 Feb 10;14:31. doi: 10.1186/1471-2180-14-31 (PMC3922988; doi:10.1186/1471-2180-14-31)
Supplement: Additional file 11 — Table of primers used in this study. [file 1471-2180-14-31-S11.docx]

**Additional file 11**. Primers used in this study

| Primer name | Sequence (5’ → 3’) | Comment |
| --- | --- | --- |
| 1465 | **GGGGACAAGTTTGTACAAAAAAGCAGGCT**GCTTGTCCTTCATTTTCGTTC | Used for amplification of flanking region of *lukF-PV* for knockout of *lukSF-PV* and recombination into pKOR1. Contains attB1 sequence at 5’ end (bold) |
| 1466 | TTTTTCCGCGGTGGGGTTCTAAGTACAACATTTCA | Used for amplification of flanking region of *lukF-PV* for knockout of *lukSF-PV* and recombination into pKOR1. Contains SacII restriction at 5’ end (underlined) |
| 1467 | TTTTTCCGCGGTTCCCCATTGAACACTTTTTG | Used for amplification of flanking region of *lukS-PV* for knockout of *lukSF-PV* and recombination into pKOR1. Contains SacII restriction at 5’ end (underlined) |
| 1468 | **GGGGACCACTTTGTACAAGAAAGCTGGGT**GCAACCGAATAAGCAGTTTTG | Used for amplification flanking region of *lukS-PV* for knockout of *lukSF-PV* and recombination into pKOR1. Contains attB2 sequence at 5’ end (bold) |
| 1842 | **GGGGACAAGTTTGTACAAAAAAGCAGGCT**CGGGGTAGGAAATTGTAGCA | Used for amplification of flanking region of *agrA* and recombination into pKOR1. Contains attB1 sequence at 5’ end (bold) |
| 1843 | **GGGGACCACTTTGTACAAGAAAGCTGGGT**AACAATTTCACACAGCGTGTTTAT | Used for amplification of flanking region of *agrA* and recombination into pKOR1. Contains attB2 sequence at 5’ end (bold) |
| 2091 | **GGGGACAAGTTTGTACAAAAAAGCAGGCT**GAATTGTCACATACTTTTTCAAC | Used for amplification of flanking region of SAA6159_00084 and SAA6159_00085 and recombination into pKOR1. Contains attB1 sequence at 5’ end (bold) |
| 2092 | **GGGGACCACTTTGTACAAGAAAGCTGGGT**GCCAACAACAGAGATGTTAACC | Used for amplification of flanking region of SAA6159_00084 and SAA6159_00085 and recombination into pKOR1. Contains attB2 sequence at 5’ end (bold) |
| 2087 | **GGGGACAAGTTTGTACAAAAAAGCAGGCT**TCCATACAAAATCCGCATCA | Used for amplification of flanking region of *hla* for knockout of this region and recombination into pKOR1. Contains attB1 sequence at 5’ end (bold). |
| 2088 | TTTTTCCGCGGTGGGAAAAAGAAGAAATGACAAA | Used for amplification of flanking region of *hla* for knockout of this region and recombination into pKOR1. Contains SacII restriction at 5’ end (underlined) |
| 2089 | TTTTTCCGCGGTGGAACCTAGCAATAGTGTTGTTG | Used for amplification of flanking region of *hla* for knockout of this region and recombination into pKOR1. Contains SacII restriction at 5’ end (underlined). |
| 2090 | **GGGGACCACTTTGTACAAGAAAGCTGGGT**CGTTCCTCAAATCTTGTACGG | Used for amplification of flanking region of *hla* for knockout of this region and recombination into pKOR1. Contains attB2 sequence at 5’ end (bold). |
| IM584 | **CCTCACTAAAGGGAACAAAAGCTGGGTACC**CCTCATTTTTCATCTCTCGTATAATTGC | Primers IM584/IM585 amplify the AB upstream flanking sequence of *psmα* for the knockout of this region. In bold, sequence complementary to pIMAY for SLIC cloning. |
| IM585 | CATTAAGATTACCTCCTTTGCTTATGAG |  |
| IM586 | *CATAAGCAAAGGAGGTAATCTTAATG*TAATTTAAGCGAATTGAATACTTAAAATTC | Primers IM586/IM587 amplify the CD downstream flanking sequence of α-*psm* for the knockout of this region. AB and CD products were joined by SOE PCR with IM584/IM587. In bold, sequence complementary to pIMAY for SLIC cloning. |
| IM587 | **CGACTCACTATAGGGCGAATTGGAGCTC**CTAGGACATGTATGTGTCTTAGTCC |  |
| IM588 | CAATTGAAAACTTAACACTGCATAACC | Chromosomal primer used to screen the *psmα* deletion. |
| IM589 | GTGATAGTTTTGATAAAGCAGAAATTTGC | Chromosomal primer used to screen the *psmα* deletion. |
| IM590 | CATTAAAATCATCAAAGCAATTGTCGACATTTTCGCAAAATAATTTAAGC | Primers IM584/IM590 amplify a 500 bp upstream and through to *psm-α4* (AB). Inserts a novel SalI restriction site. |
| IM591 | CAATTGCTTTGATGATTTTAATGATAG | Primers IM591/IM587 from *psm-α4* to yield a fragment encompassing the deletion and 500 bp down stream of *psm-α1* (CD). The AB/CD were joined by SOE PCR with IM584/IM587. |
| IM27 | **CCTCACTAAAGGGAACAAAAGCTGGGTACC**CTAACCCTCGAAATTGAAATGCTTCC | Primers IM27/IM28 amplify upstream and through to nucleotide 331 of *hla* (AB). In bold, sequence complementary to pIMAY for SLIC cloning. |
| IM28 | CAGGCCAGGCTAAACCACTTTTGTTAGC | Converts a T to a G at nucleotide 331 of *hla*, introducing a novel PstI site. |
| IM29 | CTAACAAAAGTGGTTTAGCCTGGCCTGCAGCCTTTAAGGTACAGTTGC | IM29/IM30 amplify from nucleotide 305 to downstream of the *hla* gene (CD). Underlined, nucleotide 331 modified to introduce a novel PstI site. |
| IM30 | **CGACTCACTATAGGGCGAATTGGAGCTC**CGAAAAACATCATTTCTGAAGTTATCG | The AB/CD were joined by SOE PCR with IM27/IM30. In bold, sequence complementary to pIMAY for SLIC cloning. |

Underlined: novel restriction sites; Bold: sequences complementary to the vector; Italics: region of homology for SOE PCR of AB to the CD amplimer.
